# Relationship Between Screen Usage and Speech Delay in Children Aged One to Four Years in Dubai and the Northern Emirates

**DOI:** 10.7759/cureus.73488

**Published:** 2024-11-11

**Authors:** Fatima A Alsaadi, Fathima Muzeera, Fathima Shabrina, Namra F Jafri, Raabeah F Jafri, Fatima AlOlama, Samia Farghaly

**Affiliations:** 1 Clinical Research, Dubai Medical College for Girls, Dubai, ARE; 2 Medicine and Surgery, Dubai Medical College For Girls, Dubai, ARE; 3 Surgery, Dubai Medical College for Girls, Dubai, ARE; 4 Internal Medicine, Dubai Medical College for Girls, Dubai, ARE; 5 General Practice, Dubai Medical College for Girls, Dubai, ARE; 6 Family Medicine, Child Health Sector, Primary Health Care, Dubai Health, Dubai, ARE; 7 Family Medicine, Dubai Medical College for Girls, Dubai, ARE

**Keywords:** child's screen time, electronic devices, screen time, speech and language delay, speech and language impairment

## Abstract

Background

Speech delay is the most common developmental issue among preschool children and is diagnosed when speech development is significantly lower than expected for children of the same age. As digital natives, we recognize that electronic devices can generate both beneficial and harmful outcomes for developing children in this digital age. Globally, pediatric exposure to digital screens continues to increase and is associated with undesirable effects on childhood development, particularly language skills. This study explores the influence of the quantity and quality of screen media use on the development of language skills in children aged one to four years residing in Dubai and the Northern Emirates, while also considering other factors that may contribute to speech delays.

Aim

This study aims to investigate the association between prolonged screen time and speech delay in children aged one to four years living in Dubai and the Northern Emirates. Additionally, it examines the relationship between parents' screen time and their child's screen time and speech delay, as well as the effect of multiple languages spoken at home on the child's speech development.

Methods

A cross-sectional, online-based anonymous questionnaire was conducted with the guardians of children aged one to four years from Dubai and the Northern Emirates. A total of 192 entries were collected after applying exclusion criteria. The study focused on the amount of screen time, the type of content viewed by children on various electronic devices, and its effect on speech development. Other contributing factors, including the number of languages spoken, gender, number of siblings, parents' work status, and parent-child interactions, were also examined.

Results

The study revealed that 25.5% (49 of 192 participants) had speech delays. Speech delay was most prevalent among one-year-olds, with 44.9% (22 out of 55) affected, and it was more common in males, affecting 32.7% (34 out of 104) compared to females (17%, 15 out of 88). Children who spoke more than one language had a lower prevalence of speech delay, with no delays observed in children who spoke three or more languages, and only 11.8% (10 out of 85) in those who spoke two languages. Increased screen time was linked to a higher prevalence of speech delay, with 40% (6 out of 15) of children with more than four hours of screen time affected.

Conclusion

The findings suggest that the amount of screen time is a critical factor in speech delays among young children. Prolonged screen time, particularly over four hours per day, was associated with a higher risk of speech delay. Further research is needed to explore the causal mechanisms and other contributing factors, such as parent-child interactions and socioeconomic status, that may influence speech development.

## Introduction

Speech is the production of vocal sounds and the verbal mode of communication, which includes articulation, voice, and fluency. Language involves understanding (receptive language) and expressing thoughts, feelings, and ideas (expressive language) [1,2]. Receptive language includes how the child understands language, while expressive language refers to how the child uses words to express themselves. Speech delay is the most common developmental issue among preschool children [2,3], characterized by a delay in acquiring speech sounds, using words to communicate, and understanding language lower than expected for children of the same age, in both receptive and expressive language [3]. It can vary in severity and may be caused by genetic predispositions, neurological conditions, hearing impairments, or environmental factors [4]. Recently, screen time and media consumption have been regarded as potential factors hindering children's speech development [3].

As digital natives, we recognize that televisions, cell phones, laptops, and tablets have become indispensable household items [4]. Although these devices provide beneficial tools for aiding children's development, their harmful effects, especially on younger children under the age of 7, must be fully understood and studied [3]. Globally, pediatric exposure to digital screens continues to increase and is associated with undesirable effects on childhood development, particularly language skills. In the UAE, language impairment in children is a significant concern, with an estimated 9.9% of Emirati children aged 1.5-5 years experiencing speech delays [5]. The study will look into the association between speech delay and screen usage in children aged 1-4 years and the other possible factors affecting speech.

## Materials and methods

This study adopted a cross-sectional descriptive design targeting guardians of children aged one to four years residing in Dubai and the Northern Emirates. Data were collected via an anonymous online questionnaire, available in Arabic, English, and Urdu, distributed on social media platforms and at child health centers. An information sheet clarified the voluntary nature of the study, and completing the questionnaire was considered consent. All questions in the survey were original and written by the authors using the CDC developmental milestones as the guideline. The Ages and Stages Questionnaire (ASQ), which is a standardized survey used to assess developmental milestones in young children, was used as a reference when writing the questionnaire [3]. The questionnaire included both multiple choice and open-ended questions and aimed to accurately assess a child's screen usage while taking into account confounding risk factors for speech delay, including prematurity, autism, recurrent ear infections, and family history (Appendix). The questionnaire was then translated into Urdu and Arabic to reach a wider demographic of respondents. It was reviewed by a consultant pediatrician and a family medicine consultant, and a pilot study was conducted prior to data collection. Prior approval was obtained from the Dubai Medical College for Girls (DMCG) Ethics Committee before the survey was distributed. Confidentiality and anonymity were maintained throughout the study by not including any names of children or parents in the questionnaire and by restricting access to the data collected among the authors of the research only. There was no conflict of interest between the authors.

Data analysis was performed using IBM SPSS Statistics for Windows, Version 29.0.2.0 (Released 2023; IBM Corp., Armonk, New York, United States). Descriptive statistics were used for central tendency and scatter, while the chi-square test and Monte Carlo significance were used to assess associations between variables (95% confidence interval, significant p-value <0.05). Mean or median values were compared using either independent samples t-tests (for parametric data) or Mann-Whitney U tests (for non-parametric data). Qualitative research analysis, including manual thematic extraction, was undertaken to form a theory.

Using Epi Info version 7.2, we calculated a minimum sample size of 160 with a 95% confidence interval, an estimated 11.9% prevalence of speech delay with prolonged screen usage [4], and a 5% degree of precision. A total of 258 entries were collected, with 192 remaining after applying exclusion criteria. Inclusion criteria covered children of all nationalities aged one to four years living in Dubai and the Northern Emirates, including those with autism and prematurity. Exclusion criteria included children with conditions such as deafness, mutism, Down syndrome, cerebral palsy, oropharyngeal deformities, inborn metabolic disorders, birth asphyxia, congenital infections, head injuries at birth, mental retardation, and other syndromes affecting speech.

## Results

Out of the 192 participants, 25.5% of children were found to have speech delays. The largest age group was one-year-olds (28.6%), followed by two-year-olds (25%). Males constituted 54% of the sample, with a higher prevalence of speech delay (32.7%) compared to females (17%). Most participants were non-locals (77.1%).

Age

**Figure 1:**
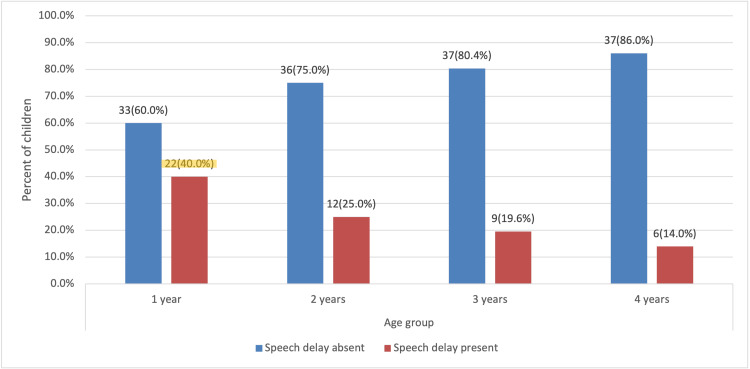
Prevalence of speech delay by age group (N=192). Chi-square tests (p=0.019).

The highest prevalence of speech delay was observed among one-year-olds (40%), with the prevalence decreasing as children grew older, dropping to 14% in four-year-olds (p=0.019) (Figure 1).

Gender 

**Figure 2:**
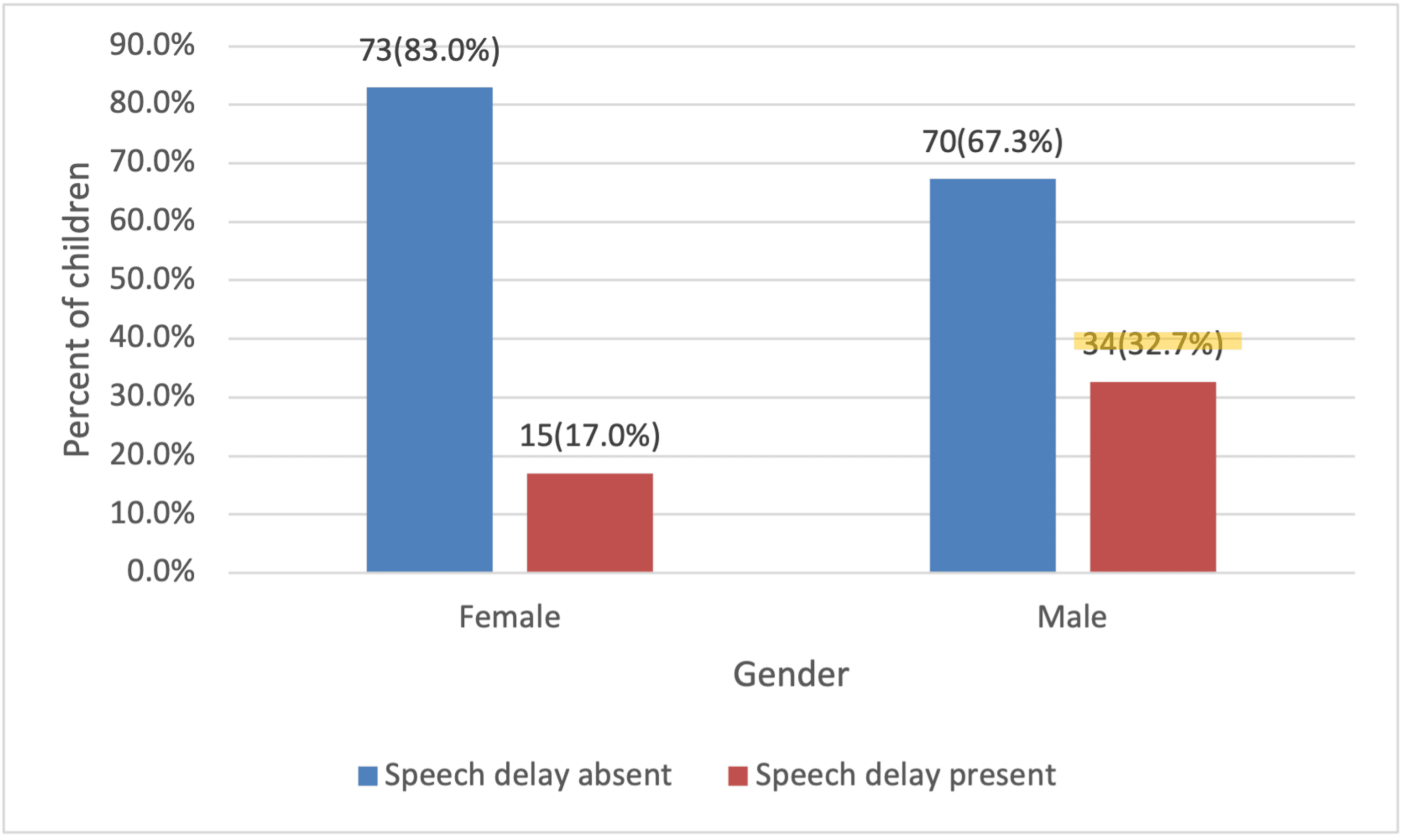
Incidence of speech delay by gender (N=192). Chi-square tests (p=0.013).

Males had a 32.7% prevalence of speech delay, 15.7% higher than their female counterparts (17%) across all age groups, a difference that was statistically significant (p=0.013) (Figure 2).

Nationality, number of siblings, and parents' employment

There was no significant effect of nationality, number of siblings, or mothers' employment on speech delay. However, children with unemployed fathers showed a higher prevalence of speech delay (66.7%) compared to those whose fathers worked part-time (41.7%) or full-time (23.7%), suggesting a potential effect of socioeconomic status (p=0.078).

Comparison with other siblings

**Figure 3:**
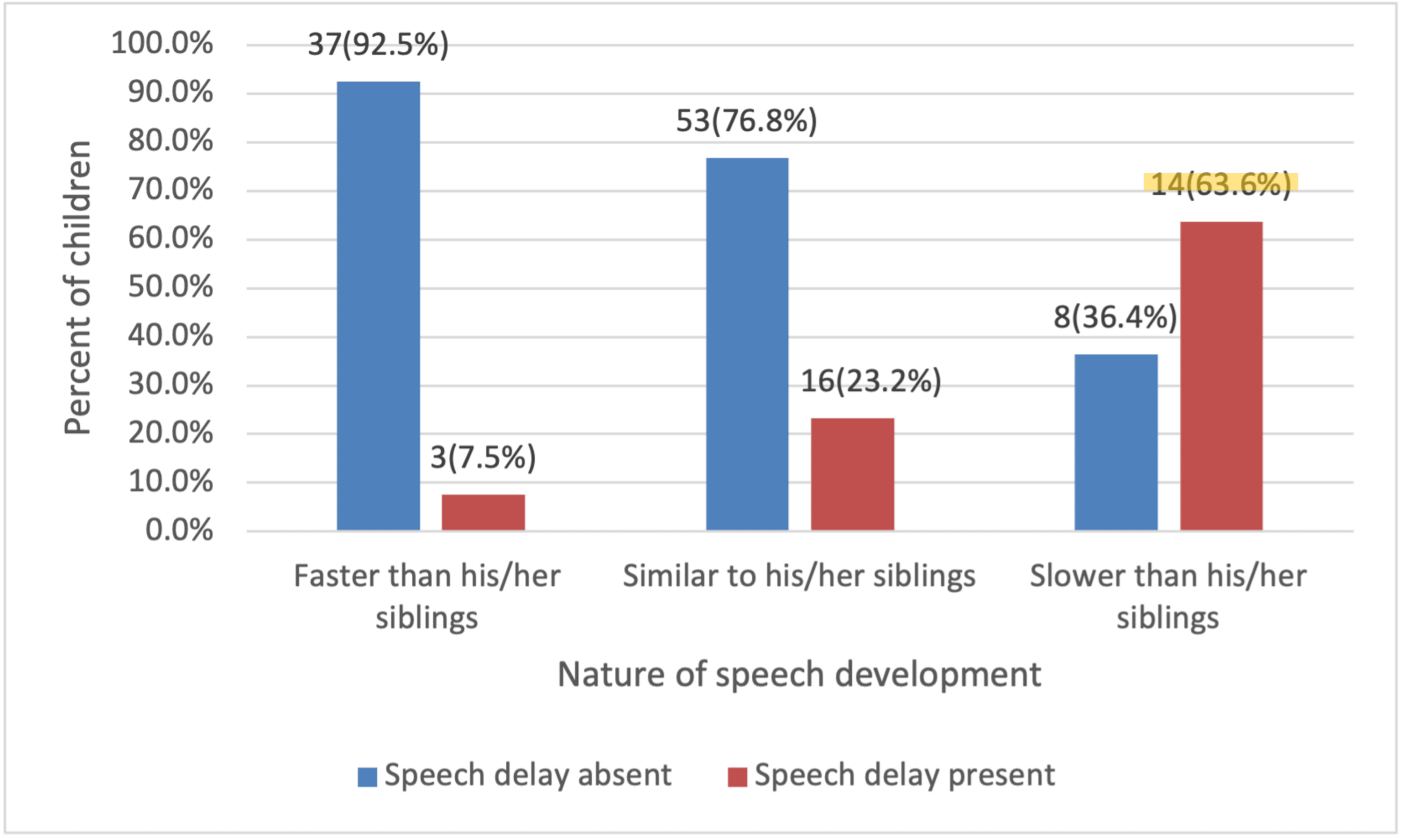
Nature of speech development in comparison with other siblings (N=131). Chi-square tests (p≤0.001).

A total of 63.6% of children with speech delay were reported to start speaking later than their siblings, while only 7.5% of children with speech delay started speaking earlier than their siblings (p≤0.001) (Figure 3).

Caretaker

Of the 192 participating parents, the majority (68%) cared for their children together. Meanwhile, 19% of children were primarily taken care of by a babysitter, and the remaining 13% were cared for by relatives. Speech delay was found in 36.4% of the children who were cared for exclusively by a babysitter. In comparison, 24.6% of children cared for by their parents and 16.7% of those cared for by relatives experienced speech delay. No significant association was found between the type of caretaker and speech delay.

Languages spoken by the child

**Figure 4:**
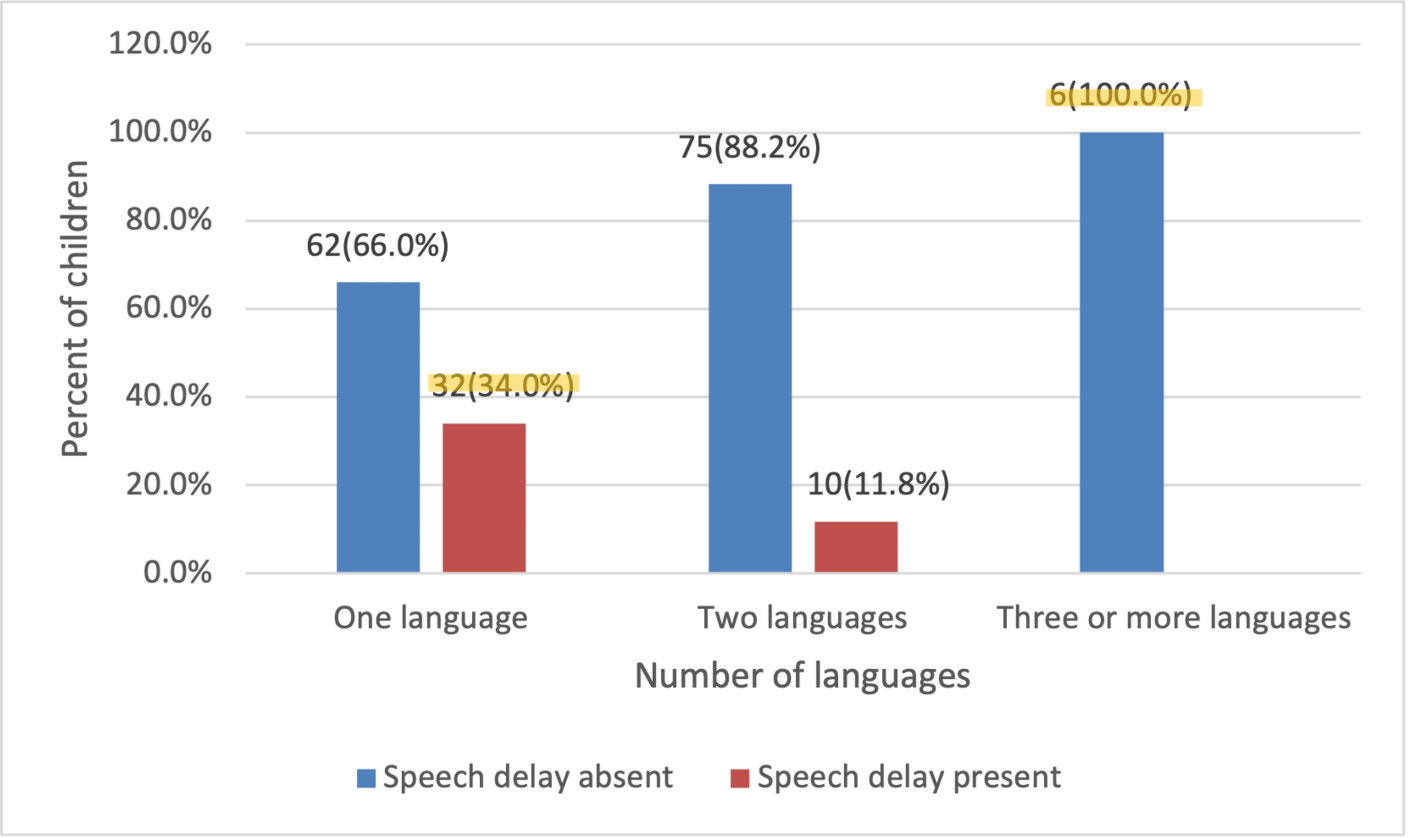
Number of languages spoken by the child and the incidence of speech delay (N=185). Chi-square tests (p=0.001).

Over half (50.8%) of the children studied spoke only one language, 45.9% spoke two languages, and the remaining 3.2% spoke three or more languages. Results showed a statistically significant relationship between the number of languages spoken and speech delay (p=0.001). The more languages a child spoke, the less likely they were to develop speech delay. Among children who spoke only one language, 34% exhibited signs of speech delay, compared to 11.8% of those who spoke two languages. None of the children who spoke three or more languages had speech delay (Figure 4).

Nursery and school

The majority (71.4%) of the children in this study did not attend nursery or school. Of the remaining participants, 17.2% attended nursery, and 11.5% were enrolled in kindergarten. No significant association was found between attending nursery or school and speech delay (p=0.110). Speech delays were observed in 21.2% of children who attended nursery and 9.1% of those in kindergarten, compared to 26.2% of children who stayed at home.

Time spent with the child

Of the parents, 45.8% reported spending all their time interacting with their children, while 45.3% spent most of their time engaging with them. The remaining 8.9% spent time with their child occasionally. There was no significant association between the time spent speaking to the child and speech delay (p=0.003). Of the children, 41.2% who spent only some time with their parents developed a speech delay, while 34.1% who spent all their time with their parents had speech delays. Only 13.8% of children who spent most of their time with their parents experienced speech delay.

Prematurity

A total of 33.3% of children with speech delay had a history of premature birth. Conversely, 66.7% of children without speech delay also had a history of premature birth (p=0.348). No significant association was found between prematurity and speech delay.

Family history

Among children with a positive family history of speech delay, 36.8% had speech delays themselves, while the majority (63.2%) did not. No significant association was found between a family history of speech delay and its occurrence in children.

Ear infections

Fifty percent of children with speech delay had a history of ear infections at least twice a year. Additionally, 30% had a history of one ear infection annually, and 24.4% had no history of infections. Notably, all (100%) children with a history of three or more infections annually did not experience speech delays. The majority (46.2%) of children with both speech delay and ear infections were less than one year old. Additionally, 25% of children with speech delay did not undergo hearing tests in early life. No significant association was found between ear infections and speech delay.

Number of hours of screen usage

A large proportion of participating parents (49.3%) reported that their child's screen time was less than two hours daily. Meanwhile, 40.4% of parents reported their child used screens for two to four hours per day, and the remaining 10.3% reported daily screen usage exceeding four hours.

**Figure 5:**
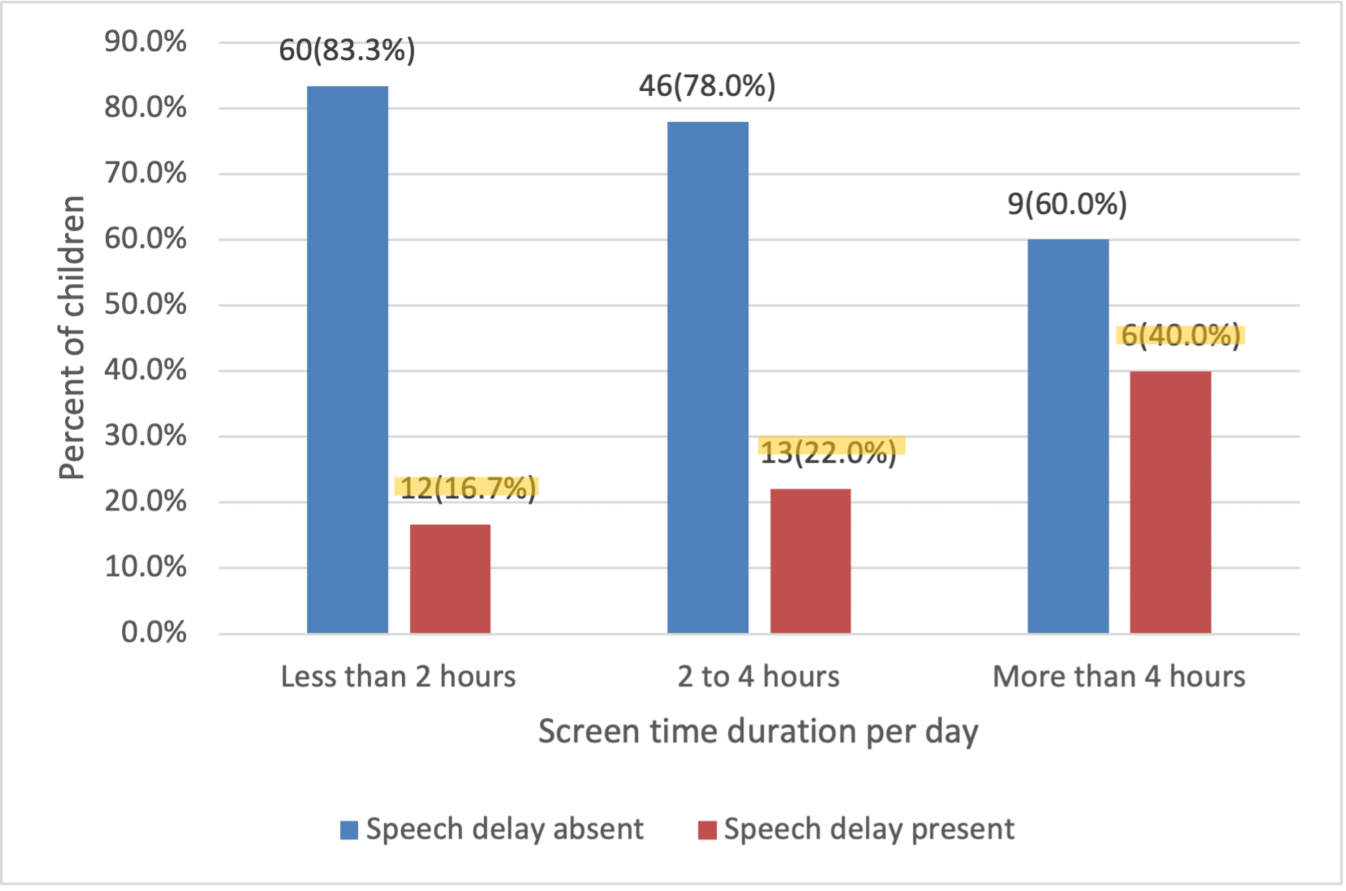
Number of hours of screen time per day and the incidence of speech delay (N=146). Chi-square tests (p=0.130).

Forty percent of children whose screen time exceeded four hours per day were found to have a speech delay. This is in comparison to approximately 22% of children who used screens for two to four hours and 16.7% of children whose screen time was less than two hours daily (p=0.13) (Figure 5).

The highest likelihood of speech delay was recorded among children who were introduced to screens earlier in life. A total of 34.4% of children who were given access to screens before the age of one year had a speech delay, while only 11.1% of those who began using screens at ages 3 to 4 showed signs of speech delay.

A decrease in the prevalence of speech delay was observed with increased parental supervision during screen use, highlighting the importance of parent-child interaction in reducing the likelihood of speech delay.

Purpose of screen usage

The majority of parents (43.2%) reported that their child used screens primarily to watch cartoons and videos, while 26% mentioned their child watched a combination of cartoons, videos, and educational content.

Children (23.3%) who watched cartoons and videos had a speech delay, followed by 22.2% of those using screens for gaming and cartoons/videos, and 21.1% for watching a combination of cartoons/videos and educational content. The association between speech delay and the purpose of screen usage was statistically insignificant (p=0.79).

Parents' screen time

Most participating parents (38%) reported using electronic devices for two to four hours daily. Meanwhile, 32.8% used devices for less than two hours, and 29.2% used them for more than four hours daily.

Speech delay was found in approximately 34.9% of children whose parents had less than two hours of screen time, 23.3% of those whose parents used screens for two to four hours, and 17.9% of those whose parents used devices for more than four hours. No statistically significant association was found between parental screen usage and children's speech delay (p=0.08).

## Discussion

The study investigated the association between speech delay and screen time among children aged one to four years in Dubai and the Northern Emirates. It is a cross-sectional study, and so it is important to note that causality cannot be inferred. Notably, it was found that children exposed to more than four hours of screen time per day exhibited a significantly higher prevalence of speech delay, with 40% affected. Additionally, the highest prevalence of speech delay was observed in one-year-olds, and males were found to have a notably higher prevalence of speech delay compared to females.

These findings resonate with previous research that has established a link between prolonged screen time and developmental issues, particularly concerning language skills. The American Academy of Pediatrics (AAP) recommends avoiding screen time for children younger than 18 months, except for video chatting, and suggests that children aged two to five years should not have more than one hour of screen time per day [6]. The findings in this study align with these guidelines, as we observed that increased screen time correlates with a higher prevalence of speech delay, especially in the youngest age group. It is essential to provide practical recommendations for parents to encourage interactive play and limit screen time in accordance with AAP guidelines to promote healthy speech development in young children. According to WHO guidelines, screen time for children less than one year of age is not recommended, while sedentary screen time is advised for no more than one hour a day for children aged two to four years [7].

Age and speech delay

The study revealed that a total of 25.5% of children had a speech delay. A similar study conducted on children under five years of age in the Indian subcontinent by Varadarajan et al. showed that 11.8% of children under two years using screens had expressive language delay, and 6.2% suffered from a delay in receptive language. Among children aged two years and older, expressive language delay was found in 24.5%, and 14.1% suffered from receptive language delay [8]. This study also revealed that, with increasing age, the prevalence of speech delay significantly reduced as children caught up with their developmental language milestones. A case-control study conducted by Al Hosani et al. in the UAE revealed that 90.3% of children between 12 and 48 months suffering from speech and language delays used electronic screen devices [9]. Al-Fadhli et al. found that 24.5% of children aged between three and five years had a speech delay in their cross-sectional study conducted in Saudi Arabia. The authors of this study attributed the increased prevalence of speech delay to excessive screen usage and the preference for indoor activities due to the country’s climate [3].

In this study, the age of first exposure to screen time significantly affected speech development, as the majority of children with speech delay were given access to screens before the age of one year (34.4%). Similarly, Kamarudin et al. revealed in their study that the first exposure to screens for children with speech delay was significantly earlier than the age of exposure of children without speech delay (p=0.002) [10]. Additionally, Karani et al., in their literature review, identified similar conclusions [11].

Quantity of screen usage

The duration of screen usage seems to play a significant role in determining its effect on language and communication. In this study, 40% of children whose screen time exceeded four hours daily had a speech delay, which is in line with other studies. Al Hosani et al. found a significant association (p<0.001) between children whose screen time averaged three to four hours daily and language problems [9]. Li et al. reported similar findings on children’s health, especially in cases of more than two hours of daily screen time [12]. Additionally, Al-Fadhli et al. found a positive relationship between language delay and daily screen time exceeding four hours (p=0.011) [3]. Another study investigated that the risk of speech delay among toddlers increased by 6.2 times with more than two hours of daily screen usage [2].

Few studies have shown a lower threshold of screen time causing speech delay. A cross-sectional study by Heuval et al. revealed that speech delay was present in children who used an electronic device for even less than 30 minutes and that each additional 30 minutes of screen time was associated with increased odds of parent-reported expressive speech delay [13].

Purpose of use

Approximately 23.3% of children who watched cartoons/videos had a speech delay, followed by those using screens for gaming as well as watching cartoons/videos (22.2%). This highlights the significance of the purpose of screen usage. A systematic review conducted by Alamri et al. found similar associations, where the use of electronic devices for interactive content and educational purposes was associated with better language and communication development in children, indicating the importance of the appropriate use of screen devices [14].

Child-parent interactions

A possible cause of speech delay could be attributed to the lack of sufficient interactions between the child and parents due to the time spent on electronic devices by both the child and the parents. Additionally, as children tend to follow the habits of their parents, the study surveyed the screen time of the parents to find a possible association. Speech delay was found in approximately 23.3% of children whose parents reported daily average screen time of two to four hours, while 17.9% were affected by a parental screen time of more than four hours. Varadarajan et al. showed that excess screen time among children was linked to the screen time of their mothers [8]. Another study by Alamri et al. reported similar results in their systematic review, showing the negative impact of increased parental screen time on their child’s speech and language development. Longer hours of daily parental screen time were found to negatively impact their child’s expressive vocabulary and grammar skills, serving as a barrier in the parent-child relationship [14]. 

Another factor to consider is the amount of time the child spends with a caretaker. In this study, 36.4% of children with speech delay were being taken care of by a caretaker. This highlights a possible effect on speech development due to the reduced time spent with parents or a lack of social interactions with a caretaker compared to a parent.

Multilingualism

The study showed that children who spoke more languages had a lower prevalence of speech delay. None of the children who spoke three or more languages had speech delays, and only 11.8% of children who spoke two languages experienced delays. This contrasts with the common belief that multilingualism might confuse children and slow down language acquisition. Consequently, exposure to multiple languages may enhance cognitive flexibility and improve language development by exposing the child to a variety of vocabulary and giving them the chance to express themselves in different ways. Similar results were found in a study by Almekaini et al. in which the proportion of children with delayed language decreased with exposure to more foreign languages [15].

Gender differences

This study revealed that males (32.7%) were more likely to experience speech delays than females (17%). These results correspond to many studies done on gender differences in speech delay. In a study conducted by Saeed et al., the results showed that male gender could potentially be a risk factor for speech delay [4]. This could be attributed to the biological, social, or cognitive factors of the child. A possible explanation is that girls are more likely to be given more discussion time and play games requiring verbal expression, while boys would be given more physical tasks and games. Adani et al. also noted that sex hormones, cerebral anatomy, and histology also play a part in speech delay and language delay [16]. Further research is needed to better understand the underlying mechanisms behind this gender disparity, as this is a mixture of complex factors. 

Confounding factors

When analyzing the relationship between speech delay and screen usage, it is crucial to account for various confounding factors. Prematurity, recurring ear infections, and a family history of speech delay are among the significant contributors to speech delay. Although no significant association was found in this study, several studies had different results. A population-based sibling-cohort study conducted in 2021 revealed that premature birth increases the risk of language delays in early childhood (1.5, 3, and 5 years), although this risk tends to diminish as children approach school age [17]. Similarly, a cross-sectional study in 2022 involving 150 children with speech delay found that a considerable portion had a history of middle ear infections (59.3%), oropharyngeal disorders (34.7%), and associated hearing problems (76%) [18]. Similarly, a cross-sectional study by Kanhere et al. identified that the medical risk factors of speech delay include birth asphyxia, seizure disorder, and oropharyngeal deformity [19]. A study by Barry et al. also highlighted that all parents of affected children had a family history of language or speech disorders, with a notable proportion (24%) also having a first-degree relative with the condition [20].

This study also showed that children whose fathers were unemployed had the highest prevalence of speech delay (66.7%), suggesting a potential effect of socioeconomic status on screen time and speech delay (p=0.078). This could be due to the possible effect on accessibility to different learning sources (books, nursery, school) or due to the busy schedule of parents who might be working multiple shifts and do not spend enough time interacting with their children. Interestingly, Chong et al., in their cross-sectional study in a tertiary center in Kuantan, Malaysia, found that household income positively correlated with the screen time of both children and parents(p=0.02) [21].

Screen time and developmental domains

Several studies have raised the awareness that increased screen time not only affects speech development but also communication skills, social skills, and problem-solving skills. In a study by Rocha et al., it was found that increased screen time negatively affected speech development, communications skills, and social and problem-solving skills, with a positive effect on fine motor skills [22]. This effect needs to be further studied and understood to produce a significant correlation. 

Conversely, few studies did not identify an effect of screen time on speech development but rather on emotional and behavioral skills only. A study by Lin et al. concluded that children who spent more time on touch screen devices were more likely to have emotional and behavioral problems but not language delay [23].

Limitations

This study relied on parent-reported data, which may introduce bias or inaccuracies. Additionally, the cross-sectional nature of the study does not allow for the establishment of causal relationships between screen time and speech delay. Longitudinal studies are needed to better understand the long-term effects of screen time on language development. 

Despite these limitations, this study highlights the need for further research into the complex factors that contribute to speech delay in young children. In particular, future studies should explore how different types of media and interaction patterns affect language development and whether interventions aimed at reducing screen time can improve speech outcomes.

## Conclusions

In this era of digital natives, pediatric exposure to digital screens is on the rise, and this trend is associated with adverse effects on childhood development, particularly concerning language skills. This study highlights the potential negative impact of excessive screen time on speech development in children aged one to four years. The findings indicate that the amount of screen time is a more critical factor in speech delays than the quality of the content being viewed. Specifically, children who engaged with screens for more than four hours a day were at a higher risk of experiencing speech delays, with boys and younger children being particularly affected.

Interestingly, the study also suggests that multilingualism may offer a protective effect against speech delays and that parental screen time is closely linked to the screen time of their children.

While these insights are valuable, further research is necessary to establish causal relationships and investigate additional influencing factors, such as the quality of parent-child interactions and socioeconomic status, in further detail. Additionally, possible future research could focus more on a longitudinal cohort study design to investigate the long-term effect of screen time on speech delay and the possible reversal of these effects in children. It is essential for policymakers, pediatricians, and parents to consider these findings and take proactive steps to reduce excessive screen time in young children, thereby fostering healthy language development.

## Appendices

Survey questionnaire

Section 1: General Information

Q- Which Emirate do you reside in?

Options: Abu Dhabi, Dubai, Sharjah, Ajman, Umm Al Quwain, Ras Al Khaimah, Fujairah

Q- What is your child’s ethnicity?

Options: Local, Non-local

Q- Which age group does your child belong to?

Options: Less than one year, One year, Two years, Three years, Four years, Five years and above 

Q- Please select the gender of your child.

Options: Female, Male

Q- How many siblings does your child have?

Options: He/she does not have siblings, One, Two, Three, More than 3

Q- Does the mother of the child work? 

Options: No, Yes, Part-time, Yes, Full-time

Q- Does the father of the child work?

Options: No, Yes, Part-time, Yes, Full-time

Q- Who takes care of your child at home? (Multiple answers)

Options: The mother and/or father, Other family relatives, A babysitter

Q- What languages does your child speak?

Options: One language, Two languages, Three or more languages

Q- Does your child go to a nursery or school?

Options: Yes my child goes to a nursery, Yes my child goes to school (kindergarten), No my child stays at home

Section ​​​​​​​2: Past Medical History & Family History

Q- Is your child diagnosed with any of the below conditions? (Multiple answers)

Options: Autism, Down's Syndrome, Deafness, Mutism/Mute, Congenital Hypothyroidism (at birth), Cerebral palsy, Metabolic disorders (IEOM), Others (e.g. other syndromes, aphasia), None of the above

Q- Did your child experience any of the following pregnancy/birth complications? (Multiple answers)

Options: Infections (during pregnancy or at birth), Cleft lip and/or cleft palate, Premature birth/prematurity, Failure to breathe at birth/lack of oxygen to the brain (birth asphyxia), Head injuries (trauma), None of the above

Q- Has your child had any previous ear infections? 

Options: Yes once a year, Yes twice a year, Yes three or more times a year, No

Q- At what age did they get the ear infections? 

Options: Less than one​ year, One​ to two years, Two to three years, Three to four years

Q- Has your child undergone a hearing test?

Options: Yes the results were normal, Yes the results were abnormal, No

Q- Is there anyone in the family and relatives who has any of the following conditions? (Multiple answers)

Options: Autism, Deafness, Mutism/Mute, None of the above

Q- Is there a family history of delay in speaking?

Options: Yes, No

Section 3: Electron​​​​​​​ic Device Screen​​​​​​​ Time

Q- Does your child use any electronic screen devices (e.g. mobile phone, laptop, etc.)?

Options: Yes, No

Q- Which of the below mentioned electronic screen device/s does your child use or watch? (Multiple answers) 

Options: iPad/tablet, Mobile phone, Television, Computer/laptop, PlayStation/Xbox

Q- On average, how many minutes/hours in a day does your child use these devices?

Options: Less than two hours, Two to four hours, More than four hours 

Q- At what age did your child first start using the above electronic screen device/s?

Options: Less than one year, One to two years, Two to three years, Three to four years

Q- What purpose is your child using the above chosen electronic device/s for? (Multiple answers) 

Options: Educational content, Watching cartoons/videos, Gaming, Social media platforms (e.g. TikTok, etc.)

Q- On average, how many hours in a day do you as a parent use electronic devices?

Options: Less than two hours, Two to four hours, More than four hours

*Sectio*n​​​​​​​ *4: Speech Development*

Q- Is your child able to speak?

Options: Yes, No

Q- How many languages does your child speak?

Options: One language, Two languages, Three or more

Q- How often do you/your partner speak to your child during the day?

Options: All the time, Most of the time, Sometimes, Never

Q- How many words can your child speak? 

Options: No words my child only coos and babbles, My child refers to me/my partner saying "mama" and "dada," My child says three words other than "mama" and "dada," My child says six words other than "mama" and "dada," My child says nine words other than "mama" and "dada," My child says more than nine words

Q- Does your child use sentences?

Options: No, Yes two-word sentences, Yes three-word sentences, Yes my child is very expressive

Q- Compared to his/her siblings, how was your child able to learn speech?

Options: Slower than his/her sibling/s, Similar to his/her sibling/s, Faster than his/her sibling/s, He/she does not have an older sibling/no siblings at all.

Section​​​​​​​: Developmen​​​​​​​tal History

Q: GROSS MOTOR SKILLS: Is your child facing any problems with his overall movements (head movement, sitting, standing, walking) according to his/her age group?

Options: Yes, No, I am not sure

Q: FINE MOTOR SKILL: Is your child facing any problems with his fine movements ( holding a toy, moving a toy, drawing with a pen, eating with a spoon) according to his/her age group? 

Options: Yes, No, I am not sure

Q: SOCIAL/BEHAVIOURAL: Is your child facing any problems with his behavior or social skills (answers when called, shows different facial expressions, plays with you or with other kids) according to his/her age group? 

Options: Yes, No, I am not sure
